# Customizable 3D Printed Implants Containing Triamcinolone Acetonide: Development, Analysis, Modification, and Modeling of Drug Release

**DOI:** 10.3390/pharmaceutics15082097

**Published:** 2023-08-08

**Authors:** Hanna Ponsar, Julian Quodbach

**Affiliations:** 1 Institute of Pharmaceutics and Biopharmaceutics, Heinrich Heine University, Universitaetsstr. 1, 40225 Duesseldorf, Germany; hanna.ponsar@hhu.de; 2 Drug Delivery Innovation Center (DDIC), INVITE GmbH, Chempark Building W 32, 51368 Leverkusen, Germany; 3 Department of Pharmaceutics, Utrecht Institute of Pharmaceutical Sciences, Utrecht University, Universiteitsweg 99, 3584 CG Utrecht, The Netherlands

**Keywords:** 3D printing, individualized therapy, implants, triamcinolone acetonide, long-term dissolution and prediction, additive manufacturing

## Abstract

Three-dimensional-printed customizable drug-loaded implants provide promising opportunities to improve the current therapy options. In this study, we present a modular implant in which shape, dosage, and drug release can be individualized independently of each other to patient characteristics to improve parenteral therapy with triamcinolone acetonide (TA) over three months. This study focused on the examination of release modification via fused deposition modeling and subsequent prediction. The filaments for printing consisted of TA, ethyl cellulose, hypromellose, and triethyl citrate. Two-compartment implants were successfully developed, consisting of a shape-adaptable shell and an embedded drug-loaded network. For the network, different strand widths and pore size combinations were printed and analyzed in long-term dissolution studies to evaluate their impact on the release performance. TA release varied between 8.58 ± 1.38 mg and 21.93 mg ± 1.31 mg over three months depending on the network structure and the resulting specific surface area. Two different approaches were employed to predict the TA release over time. Because of the varying release characteristics, applicability was limited, but successful in several cases. Using a simple Higuchi-based approach, good release predictions could be made for a release time of 90 days from the release data of the initial 15 days (RMSEP ≤ 3.15%), reducing the analytical effort and simplifying quality control. These findings are important to establish customizable implants and to optimize the therapy with TA for specific intra-articular diseases.

## 1. Introduction

Individualized therapy is becoming increasingly important to increase therapy efficiency and reduce adverse drug reactions [1]. Adaptions of medicinal products for certain diseases considering patient-specific conditions like gender, age, body mass, comorbidities, and genetic heterogeneity are required. Also, the customization of drug release to meet pharmacodynamic or pharmacokinetic requirements is an emerging topic [2,3]. In this context, the dosage form itself must be considered. Drug release individualization is especially important for long-acting dosage forms, i.e., implants.

Drug-loaded polymeric implants as parenteral depot systems represent an effective option for certain active pharmaceutical ingredients (APIs), with restrictions for oral applications (e.g., first-pass metabolism, low bioavailability, and the need for prolonged or targeted drug delivery) [4]. Drugs can be released at a predetermined rate over an extended period of time within the therapeutic window. In addition to systemic therapy via subcutaneous injection of mostly cylindrical rods (e.g., Nexplanon^®^ for contraception), they are suitable for targeted local applications. Simultaneously, the anatomical structures can be replaced or supported. The targeted and prolonged application can result in a reduced administered drug amount, thereby reducing side effects and improving patient adherence as the frequency of administration is strongly decreased [4,5]. Compared to oral or injectable depot systems, e.g., crystal suspensions or microspheres, the characteristics of implants offer major benefits in the treatment of cancer, dental, orthopedic, post-operative local antibiotic, cardiovascular, and rheumatic therapy [6,7]. Marketed medicinal products for these intents are rare. Gliadel^®^ wafers (compressed-molded platelets containing carmustine) applied in the brain cavity after tumor removal or CiproScrew^®^ for fracture fixation while simultaneously preventing post-surgical infection are two of the few medicinal products.

However, current implants and manufacturing options, mostly hot-melt extrusion and injection molding for solid implants, do not offer individualization of dosage, drug release, and shape to anatomical structures. Another challenge is the on-demand availability during clinical routine operations [6]. Advances in new technologies are, therefore, mandatory to overcome these limitations and enable patient centricity.

Three-dimensional printing offers a vast potential to create more sophisticated implantable devices for the adaption of drug dosage and release rate control, as well as individualization for patient conditions and anatomical features. Fused deposition modeling (FDM) is of great interest in this matter due to its versatile possibilities of customization on a low-cost basis [8,9]. FDM is an extrusion-based technology, where a thermoplastic polymeric drug-loaded strand (filament) is extruded from a heatable nozzle (hotend). The molten strand is then printed on a temperature-controlled build plate. Using x, y, and z movements of the print head and/or the build plate, the final drug-loaded 3D printed drug delivery system (DDS) is created in a layer-by-layer fashion. Compared to other existing 3D printing technologies, it represents a promising approach to producing individualized dosage forms on demand at the point of care using industrially manufactured filaments [10]. A high variability in complex shapes can be realized based on computer-aided design (CAD) models. This is especially beneficial in terms of individual shape adaptions to body structures or cavities with high anatomical variability. The data for processing can be obtained from medical imaging technologies, such as computed tomography or magnetic resonance imaging [11]. This was already demonstrated for drug-free dental or orthopedic prostheses for anatomical reconstruction [12,13]. Dosages can be adapted based on the size of the geometry or the inner structure and drug release via modification of the surface area to volume ratio [9,14,15,16]. Also, multi-drug and multi-formulation devices for oral applications have been reported in the literature [9,17,18]. Until now, only a few researchers have investigated 3D printed implants to evaluate the applicability and performance of customized therapy [19]. Stewart et al. [20] and Allen et al. [21] investigated the opportunities for drug release modification using different reservoir types and varying channel diameters, respectively. API combinations were investigated in the context of post-surgical infections and the simultaneous facilitation of cell or bone growth [22,23].

Thus, further studies are required to evolve the capability of 3D printing for individualized parenteral therapy for clinically relevant APIs. In this study, triamcinolone acetonide (TA) was used as the model drug. TA is a synthetic glucocorticoid with low oral bioavailability [24], limiting its applications to topical or parenteral administration. TA is, among others, indicated for rheumatic diseases as it has anti-inflammatory activity [25,26]. For intra-articular (i.art.) applications, crystal suspensions are available. However, the frequency of administration is limited to three injections per year (minimum treatment intermissions of six weeks) [27] due to systemic side effects caused by rapid systemic absorption. This impedes pain complaint management and reduces patient compliance. Additionally, the residence time of TA within the articular cavity is limited to three to four days [28,29] because of the fast trans-synovial efflux [30]. The clinical benefits (pain relief) of the therapy can only be maintained for up to four weeks and decrease as the treatment time progresses [31,32].

Consequently, there is a need to improve therapeutic efficiency and patient compliance with the i.art. application of TA. To overcome the limitations and drawbacks of the currently available therapy options, 3D printed shape-adaptable implantable devices might be beneficial. Comparative clinical studies of a recently approved TA-loaded PLGA-based microsphere formulation (Zilretta^®^; research code FX600) with extended release characteristics showed an increase in residence time of up to three months and a reduction in plasma peak levels [28,33,34]. Therefore, therapy efficiency related to pain, stiffness, and physical functions compared to a crystal suspension significantly increased [28,35,36]. However, the opportunities for individualization with microspheres related to shape adaption and drug release modifications are limited.

The aim of this study is, therefore, the development of a novel customizable TA-loaded modular i.art. implant using the FDM 3D printing technology. Besides the individualization of dosage and drug release independent of the implant shape, prediction of the release rate in vitro should be possible. Several studies reported different implant structures to modify the drug release [37,38,39,40,41,42]. Hilgeroth et al., for example, provided an FDM-printed grid using a triblock copolymer of Styrene–Isobutylene–Styrene as a slow-release system for TA [43]. However, a concept enabling shape, dosage, and drug release control simultaneously, in combination with the prediction of the latter, has not been reported. Ultimately, the results of this study shall be beneficial for the widespread application of customized drug-loaded implants.

## 2. Materials and Methods

### 2.1. Materials

For the production of drug-free (F1) and TA-loaded (F2) filaments, powder blends of TA (micronized, Caelo, Hilden, Germany), ethyl cellulose (EC, Aqualon N10, Ashland, OH, USA), hypromellose (HPMC, Metolose 60SH 50, Shin Etsu, Tokyo, Japan), fumed silica (Aerosil^®^ 200 VV Pharma, Evonik, Darmstadt, Germany), and the liquid plasticizer triethyl citrate (TEC, Jungbunzlauer, Basel, Switzerland) were used. The compositions are presented in Table 1.

### 2.2. Methods

#### 2.2.1. Hot-Melt Extrusion of Filaments

The powder blends (500 g) were fed gravimetrically (KT 20, K-Tron Soder, Zurich, Switzerland) into a tempered barrel of a 16 mm 40 D co-rotating twin-screw extruder (Pharmalab HME 16, Thermo Fisher Scientific, Dreieich, Germany) and extruded from a 1.85 mm die. The liquid plasticizer was fed using a micro annular gear pump (MZR 7205, HNP Mikrosysteme, Schwerin, Germany) equipped with a 0.12 mm nozzle. The following screw configuration was used (gear-to-die): 8 D conveying elements (helix of 3/2 L/D); 12 D conveying elements (helix of 1 L/D); 4 × kneading discs 30° (1 D); 4 × kneading discs 60° (1 D); 10 D conveying elements (helix of 1 L/D); 4 × kneading discs 30° (1 D); 4 × kneading discs 60° (1 D); 10 D conveying elements (helix of 1 L/D). The extrusion settings were a powder feed rate of 5 g/min, a screw speed of 20 rpm, and an extrusion temperature of 190 °C (the exact temperature gradient can be found in Table S1). Behind the die, the extruded filaments were stretched to the target diameter of 1.75 mm using a winder (Model 846700, Brabender, Duisburg, Germany) with a haul-off speed of 2.1 m/min. The filaments were cooled on a static conveyor belt between the extruder and the winder. The diameter was monitored inline using a laser-based measurement module (Laser 2025 T, Sikora, Bremen, Germany) placed between the cooling section and the haul-off unit of the winder.

#### 2.2.2. Triamcinolone Acetonide Assay of Filaments

The TA content was measured using HPLC (LaChrome System, Hitachi, VWR, Darmstadt, Germany). A method with isocratic elution modified from the literature [44,45] was chosen. The HPLC consisted of an autosampler (L-2000), an oven (L2300), and a UV detector (L2400). A Eurospher II 100-5 C18A with an integrated precolumn (150 mm × 4.6 mm) served as the stationary phase. The mobile phase consisted of acetonitrile and purified water (40/60 *v*/*v*), and the flow rate was set to 1.0 mL/min. An oven temperature of 40 °C was used. The detection wavelength was 241 nm. For the content analysis of filaments, sections of approximately 200 mg over the whole filament length (n = 6) were dissolved in ethanol 90% [*w*/*w*] at 40 °C using sonification. Colloidal solutions were filtered prior to further dilution through a 0.45 µm polypropylene membrane filter (VWR, Leuven, Belgium). To determine the residual TA content of intact EC filament pieces (n = 3) after the drug release studies, the same analytical procedure was applied. The method was validated with respect to linearity, repeatability, intermediate precision, specificity, and recovery according to the Reviewer Guidance (1994) in alignment with the ICH Guideline Q2 [46,47].

#### 2.2.3. Design and 3D Printing of TA-loaded Implants

**Figure 1:**
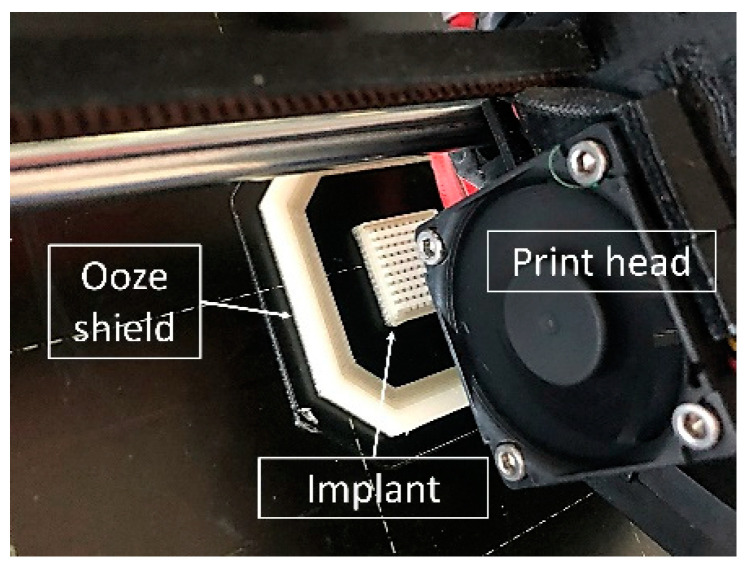
Picture of the dual 3D printing process of two-compartment implants.

Two-compartment implants were designed using the CAD software Inventor^®^ Professional 2019 (Autodesk^®^, San Rafael, CA, USA). As the slicing software, Simplify3D^®^ software (version 4.0) (Cincinnati, OH, USA) was used. Printing was performed using the Prusa i3 MK3S FDM printer (Prusa Research, Prague, Czech Republic). The printer was equipped with a multi-material unit (MMU) 2S, enabling a dual-component print of the drug-free shell and drug-loaded network inlay. An ooze shield was printed on every layer around each implant to prevent cross-contamination after each automatic filament change (Figure 1). Printing temperature was 185 °C, layer height was 200 µm, and nozzle diameter was 400 µm. Infill density was 100%. The rectilinear fill pattern was printed at 45° for the drug-free shell and 90° for the drug-loaded network inlays. More detailed printing settings can be found in the supplementary material (Table S2).

#### 2.2.4. Drug Content Determination of Two-Compartment Implants

For the evaluation of the drug release of two-compartment implants, TA loading was determined based on the mass of the drug-containing inlays and the TA content in filament F1. To determine the mass of the drug-loaded inlay non-destructively, 10 drug-free shells were printed separately for each implant size. The shells were weighed on an analytical balance, and the mean mass of the implant shell was subtracted from the total mass of the respective two-compartment implant used for long-term dissolution studies. The content of each implant was then calculated based on the calculated masses, and the TA content of the filament F1 was determined.

#### 2.2.5. Determination of the Implant Surface Area Using X-ray Microcomputed Tomography

The surface area and dimensions of the drug-loaded networks of the two-compartment implants were determined using X-ray microcomputed tomography (CT-COMPACT 130, ProCon X-ray, Sarstedt, Germany, n = 1). The settings were as follows: 1300 projections, 20 µm voxel size, 80 kV acceleration voltage, and 187 µA current. The reconstruction was performed using VG Studio software 3.0.1 (Volume Graphics, Heidelberg, Germany). Avizo Fire software (9.0.1, Thermo Fisher Scientific, Waltham, MA, USA) was used for image processing (visualization, determination of surface area, and dimensions).

#### 2.2.6. In Vitro Long-Term Dissolution Analysis

**Figure 2:**
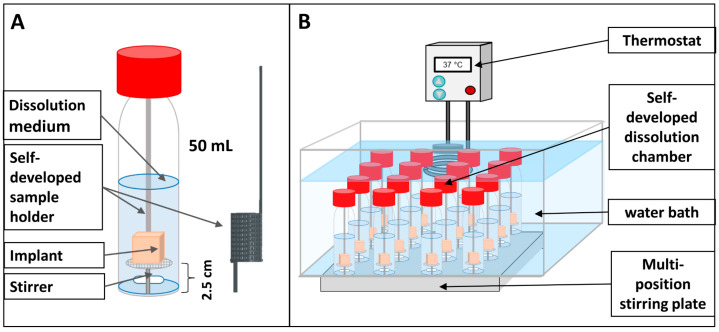
Schematic illustration of the developed dissolution setup for long-term dissolution studies of parenteral DDS. (**A**) Dissolution chamber setup equipped with an in-house built sample holder for parenteral DDS and 3D printed filament basket (black). (**B**) Dissolution setup for multi-sample drug release analysis.

The 3D printed implants and the drug-loaded filaments were subjected to long-term dissolution studies under sink conditions (n = 3). The analysis was performed in a self-developed dissolution setup consisting of glass flasks with watertight closure equipped with a magnetic stirrer placed in a water bath on a multiposition stirring plate (Labomag (4 × 4), SHP Steriltechnik, Haldensleben, Germany) (Figure 2). Implants were fixed upright on self-produced sample holders with cyanoacrylate-based, solvent-free glue (UHU^®^ Alleskleber super, UHU GmbH & Co. KG, Bühl, Germany) equipped with a spacer of 2.5 cm length to enable reproducible test conditions. Filament sections were placed in 3D printed baskets (3 × 2.5 cm sections per basket; Figure 2A). Tests were performed under two different conditions to maintain sink conditions. During weekdays, 50 mL HEPES buffer pH 7.4 at 37 ± 0.5 °C with a stirring speed of 200 rpm was used. Over the weekends, the volume was increased to 75 mL. Samples of 5–30 mL (depending on the sampling frequency) were withdrawn manually during the initial 10 days using a one-channel pipette (Research^®^ Plus, Eppendorf, Ulm, Germany). To maintain sink conditions, a complete media change was performed at sampling intervals of ≥12 h. Samples were filtered through a 0.45 µm polypropylene or nylon membrane filter (VWR, Leuven, Belgium) prior to further analysis.

Quantification was performed by HPLC using the method described in Section 2.2.2. We found that TA degrades into impurity C (TA-21 aldehyde hydrate) in commonly used pharmaceutical buffer systems. Therefore, extensive studies were performed to investigate the degradation. In the HEPES buffer, degradation could not be prevented but could at least be strongly reduced. Therefore, it was selected as the dissolution medium in this study. As both substances, TA and impurity C, showed a similar extinction coefficient in preliminary studies, the released TA amount was calculated based on the sum of the peak areas of TA and detected impurity C. The TA recovery of stressed samples (n = 3 varied between 95.18 ± 0.78% (after three days) and 97.85 ± 1.30% (after four hours)). This was considered acceptable for a discriminative analysis of different 3D printed implants. The relative drug release from intact implants was calculated based on the drug load determined, as described in Section 2.2.4. The remaining TA content of the filaments was determined as described in Section 2.2.2. The sum of the released TA from the filaments and the residual TA concentration was considered as the total TA filament content.

#### 2.2.7. Drug Release of Volon^®^ A 10-5 mL (TA Crystal Suspension)

The dissolution behavior of Volon^®^ A 10-5 mL (TA crystal suspension, Dermapharm, Grünwald, Germany) was examined using USP apparatus II (DT 700, Erweka, Germany) at a stirring speed of 100 rpm. A study in the dissolution setup described in Section 2.2.6 for implants was not possible due to the low TA solubility in the medium (20.27 ± 0.41 mg/L at 37 °C, n = 6) and limitations in the accuracy of sampling from the crystal suspension at reduced quantities. To maintain sink conditions and allow for a discriminative analysis compared to implants, a dose of 6 mg TA was investigated in 1000 mL HEPES buffer at pH 7.4 37 ± 0.5 °C [48]. A total of 0.6 mL of TA crystal suspension (n = 6 mg TA) was transferred to a vessel using a tuberculin syringe equipped with a luer-lock connection cannula 0.90 × 40 mm. To allow reproducible sampling, 0.8 mL samples were withdrawn at predetermined time points using an automatic syringe pump (Legato 111, KD Scientific, Holliston, MA, USA) at a rate of 30 mL/min and replaced with fresh medium. Syringes were connected to 10 µm pore-sized glass frites to reduce or avoid the removal of undissolved crystals. Prior to content analysis via HPLC (see Section 2.2.2), samples were filtered through a 0.45 µm nylon filter (VWR, Belgium). The TA amount after achieving the plateau was considered the total API content, as the removal of undissolved TA crystals during sampling could not be excluded with certainty.

#### 2.2.8. Fit by the Korsmeyer–Peppas Model

Dissolution curves were analyzed using the Korsmeyer–Peppas approach (Equation (1)) [49]:

(1) $$\frac{M_{t}}{M_{\infty }}=kt^{n}$$

where $\frac{M_{t}}{M_{\infty }}$ is the released API amount at time *t*, *k* is a constant indicative of the geometry, and *n* represents the diffusional exponent representative of the underlying release mechanism. The double logarithmic depiction leads to the linearization of Equation (2).

(2) $${log}_{10}\frac{M_{t}}{M_{\infty }}={log}_{10}\;k+n\cdot {log}_{10}t$$

Values between 5% and 60% drug release were used for the fitting. The diffusional release exponent was obtained as the slope of the linear regression from Equation (2) to determine the respective release kinetic.

#### 2.2.9. Fitting of Dissolution Curves

Drug release profiles were fitted to the Higuchi, Peppas–Sahlin, and Weibull mathematical models according to the approach of Windolf et al. for non-erodible matrices (up to 60% of drug release). The exact procedure and model description can be found in [50]. The NLFit application Origin (Pro 2019/2020, OriginLab Corporation, Northampton, MA, USA) was used to fit the different mathematical models. For iterations, the Levenberg–Marquard algorithm was applied to determine the non-defined constants of the respective model.

#### 2.2.10. Prediction of Drug Release Using the Higuchi Model

The prediction of the in vitro data from implants using the Higuchi model was performed according to Korte and Quodbach [15]. The Higuchi model for homogenous, porous, and planar polymer matrices was described using Equation (3), where *Q* represents the released API amount at time point t (*M_t_*) per unit area (*A*) [51], *D* is the diffusivity of the drug inside the polymer matrix, *ε* is the porosity of the matrix, *τ* is the tortuosity of the capillary system, *C*_0_ is the concentration at time 0, and *C_s_* is the saturation concentration in the polymer.

(3) $$Q=\frac{M_{t}}{A}=\sqrt{\frac{D\varepsilon }{\tau }(2C_{0}-{\varepsilon C}_{s})C_{s}t}\;$$

Equation (3) can be simplified to Equation (4), where *D_k_* combines the diffusional coefficient, porosity, and tortuosity of the porous matrix, as well as the initial and saturation concentrations in the polymer [52].

(4) $$M_{t}=D_{k}A\sqrt{t},\;\mathrm{with}\;D_{k}=\frac{Q}{\sqrt{t}}$$

Using *D_k_* of a given implant with the same strand width and predetermined surface area of the implant of interest, the drug release was predicted.

To predict the drug release based on the data of the initial 15 days of the respective implant, the percentage of released TA (until 60%) was plotted against $\sqrt{t}$. A linear regression of the resulting data was used to predict the release curve of the remaining time (75 days).

#### 2.2.11. Root Mean Square Error of Prediction (RMSEP)

The goodness of prediction of the drug release was assessed using the RMSEP according to the following equation:

(5) $$RMSEP=\sqrt{\frac{\sum_{i=1}^{n}{\left(y_{i}-{\hat{y}}_{i}\right)}^{2}}{n}}$$

where *n* is the number of samples *i*, $y_{i}$ is the observed, and ${\hat{y}}_{i}$ is the predicted value for each time point.

## 3. Results and Discussion

### 3.1. Development of a Modular Implant Concept Using Computer-Aided Design (CAD)

#### 3.1.1. Development of a General Customizable Modular Implant Design

The first aim of the present study was the development of a widely applicable, customizable modular implant system allowing for individual adaption of dosage, drug release, and shape to patient requirements. In the case of the i.art. application of the model drug TA, the residence time in different joints up to multiple months should be enabled to increase the therapeutic efficiency compared to currently marketed depot injections. However, the concept is applicable to various parenteral application sites where shape adaption appears reasonable. Dosage and drug release must be independently individualized to treat specific patient conditions, as every change in shape would affect those conditions and consequently reduce the degree of customization [14]. It was already shown that a combination of local drug application and anatomical defect replacement or adaption reduced therapy duration and the medical outcome in orthopedic surgeries [53]. To the best of our knowledge, such a concept has not been covered in the literature using FDM.

**Figure 3:**
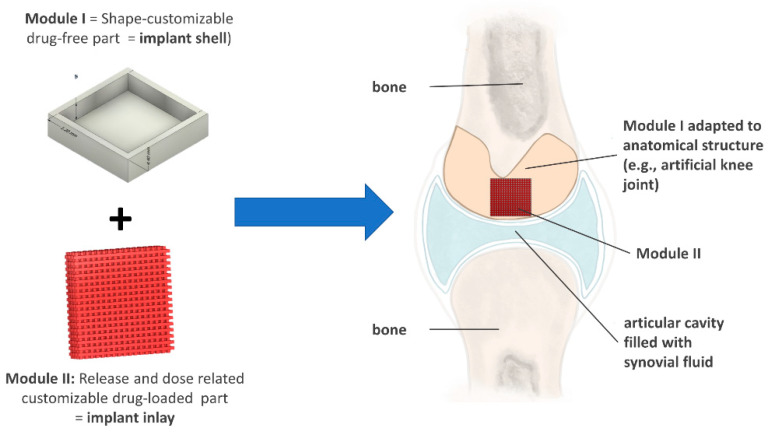
Modular implant concept and application in the articular cavity with shape adaptation to the anatomical body part.

The newly developed modular implant consists of two components: a drug-free shell (module I), which surrounds a drug-loaded inlay (module II), as schematically depicted in Figure 3. The drug-loaded part (module II) can be systematically adjusted to individualize dose and drug release. The drug-free module (module I) can be adapted independently to anatomical structures, such as joints or bones. Module I enables a unidirectional drug release from module II to the site facing the body fluid. Consequently, the implant is not limited in thickness or shape, and the therapy can be localized precisely without affecting the surrounding tissues.

The drug-loaded part was designed as an adaptable network structure using CAD. To systematically modify the drug release, two degrees of freedom, strand width and pore size, were identified. Hence, the drug-eluting surface area and diffusion path lengths were varied, and the impact of drug diffusion into the medium (not rate-limiting) could be investigated. The opportunities for customization using FDM should be evaluated, and optimal strand width/pore size combinations for the intended use should be identified.

In the case of TA, a targeted drug delivery over three months was aimed at reducing the number of surgeries while optimizing therapy efficiency. Preliminary dissolution experiments of the filaments with a diameter of 1.72 mm showed that TA release was too slow and had to be accelerated to achieve the target dissolution behavior. This can be achieved by increasing the drug-eluting surface area by decreasing the diameter of the printed network strands compared to the filaments.

**Figure 4:**
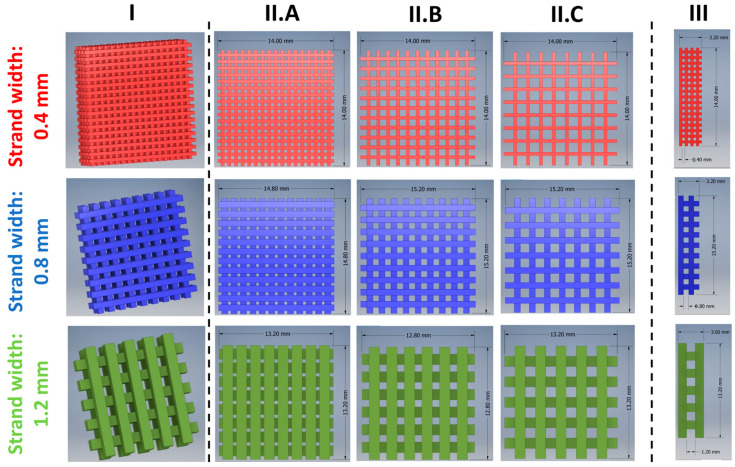
Drug-loaded network inlays designed using CAD software to modify the TA drug release. (**I**) 3D CAD model; (**II**) top view; (**III**) side view (z-direction). Strand widths 0.4 mm (red), 0.8 mm (blue), and 1.2 mm (green) were combined with pore sizes of 0.4 mm (**A**), 0.8 mm (**B**), and 1.2 mm (**C**), respectively.

The designed network structures (module II) used to modify the TA release from implants are depicted in Figure 4. A quadratic form was chosen to simplify the experimental design. However, in later applications, the shape can be freely modified. The smallest possible strand width was 0.4 mm. Strand widths of 0.4 mm (red), 0.8 mm (blue), and 1.2 mm (green) were selected and combined with three pore sizes (0.4 mm, 0.8 mm, and 1.2 mm) in the x- and y-directions, respectively.

The pore size in the z-direction was always equal to the strand width. In this study, different implants were named according to the following convention: YY × ZZ. YY indicates the strand width and ZZ is the pore size in mm. The dimensions and drug loads of the inlays are shown in Table 2. The true dimensions of the strand width were determined for implants of 0.4 × 0.4, 0.8 × 0.8, and 1.2 × 12 and can be found in the supplementary material (Table S2). An equal size of the implants with a strand width of 0.8 and 1.2 mm with varying pore sizes could not be realized (Table 2). They differed slightly, as otherwise the distances between printed strands and module I would have varied, potentially impacting dissolution.

The created network was embedded in an impermeable shell as a surrogate for the shape-adaptable part (1.2 mm thickness), as depicted in the technical drawing (Supplementary Material, Figure S1), enabling the targeted unidirectional TA release.

The size of the implants designed in this study is rather large to facilitate modifications and characterization. Consequently, the application of the presented geometries in specific joints is likely not possible due to size restrictions. However, the results of this study allow for the rational design of implants for specific applications.

#### 3.1.2. Production and Physical Properties of Two-Component Implants

For the printing of implants, filament formulations F1 and F2 were used for the drug-loaded inlay and the drug-free shell, respectively. For both parts, a low-substituted EC was selected as a thermoplastic, inert, biocompatible, and non-biodegradable pharma-grade polymer. Due to its properties, it enables a strong diffusion-controlled sustained release behavior of the model API out of the matrix, as necessary for a parenteral depot DDS [54,55]. In addition, it is suitable as a surrogate polymer for the shape-adaptable part to evoke unidirectional drug release, as drugs will not or only minimally diffuse through EC when another sink reservoir (synovial liquid) is available. EC showed good performance during HME and FDM printing [56,57,58,59,60]. A low-viscosity HPMC type was added as a pore former to F1 to accelerate TA release from the strongly sustained EC matrix of the implant [61].

**Figure 5:**
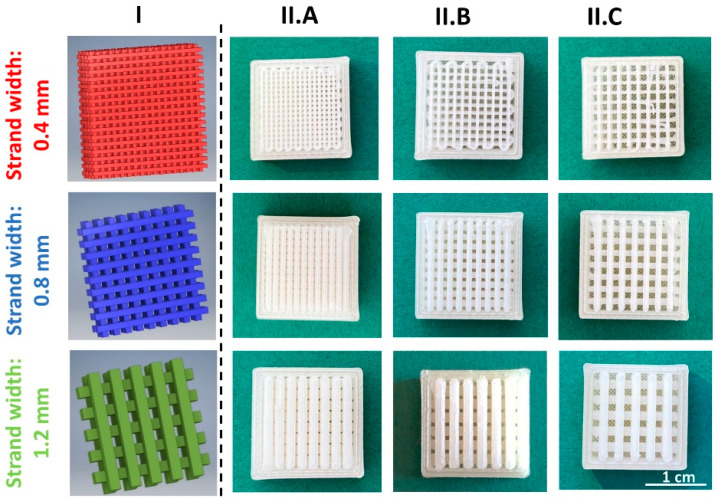
Overview of 3D printed implants, consisting of the drug-free impermeable shell and the TA-loaded network inlay. (**I**) 3D CAD model of the network inlay; (**II**) pictures of the printed implants with strand widths of 0.4 mm (red), 0.8 mm (blue), and 1.2 mm (green) combined with a pore size of 0.4 mm (**A**), 0.8 mm, (**B**) and 1.2 mm (**C**), respectively.

As shown in Figure 5, all designed implants were printable and of good quality. The production of such fragile network structures is not possible with traditional pharmaceutical manufacturing methods, such as injection molding [8].

The physical characteristics of the printed implants were determined. Complete implants consisting of an outer shell and an inner network showed good mass uniformity with a relative deviation of ≤2.6% (n = 3). The mass of the drug-loaded module II was determined as described in Section 2.2.4.

**Figure 6:**
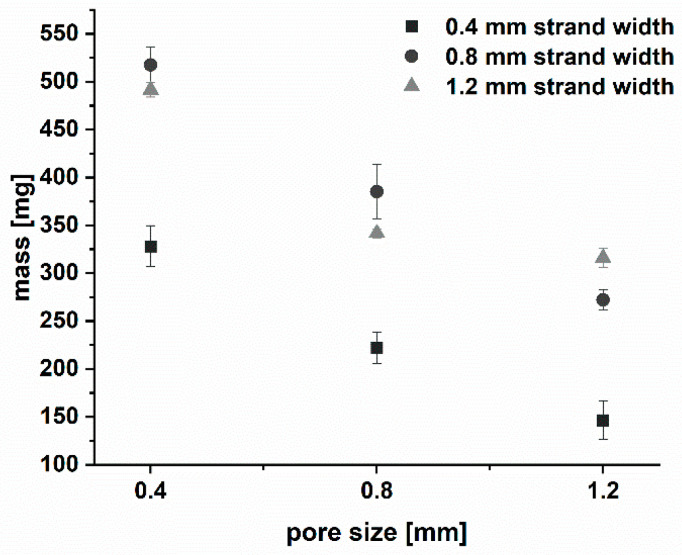
Plot of mass vs. pore size of the 3D printed drug-loaded implant inlays for the respective strand width of the network (n = 3; mean ± s).

The masses increased with increasing strand width and decreasing pore size and varied between 146 and 517 mg (Figure 6 and Table 2). To keep the implant sizes comparable, the network dimensions of the implants with a strand width of 0.8 mm and 1.2 mm differed slightly depending on the strand width and pore size combination (Table 2), as described above. Consequently, only for implants with strand widths of 0.4 mm, a linear correlation was observed (R^2^ = 0.998).

The deviations for module II varied (±4.74 to 21.53 mg), depending on the strand width and likely induced by the method for mass determination (Section 2.2.4). The deviations may be caused by the manual deduction of the weight of the shell, as described above. This assumes a constant weight of the shells, which is likely not the case.

Clinically relevant TA doses between 13 and 46 mg were realized (Table 2), demonstrating the benefit of 3D pinting to adapt the dosage without changing the formulation. As described in Section 2.2.4, the content of the drug-loaded modules was calculated based on the mass of the drug-loaded network and the recovered drug content in the filament. The target content in the filaments was 10%, but only the relative amount of 88.58 ± 2.24% was recovered. This reduction in content may be due to the adhesion of TA to the walls of the mixing vessel and the gravimetric feeder. Thermal degradation was investigated but could be ruled out.

**Figure 7:**
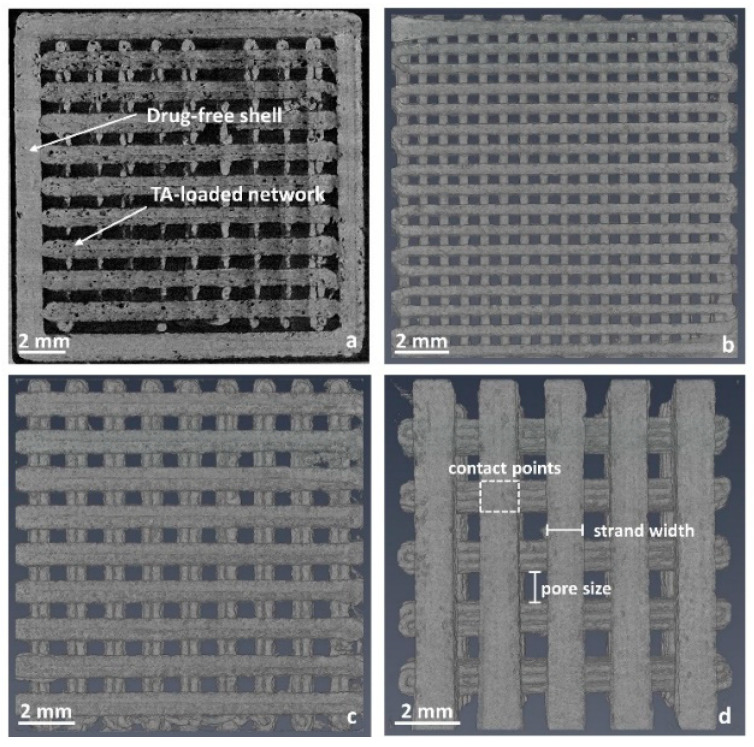
Volume renderings of 3D printed implants. (**a**) Complete implant (shell + network; 0.8 × 0.8 mm); (**b**) drug-loaded network 0.4 × 0.4 mm; (**c**) drug-loaded network 0.8 × 0.8 mm; (**d**) drug-loaded network 1.2 × 1.2 mm.

X-ray computed tomography was used for the determination of the true surface area of the implants. A selection of images is shown in Figure 7. In Figure 7a, a complete implant (shell and network) is exemplarily depicted. To obtain mostly quadratic network strands, a printing layer height of 0.2 mm was chosen. Strands of 0.4 mm width consisted of two printed layers and an individually printed strand (Figure 7b). As the width of one single printed strand was predetermined by the used nozzle diameter of 0.4 mm, the networks 0.8 mm and 1.2 mm consisted of two (Figure 7c) and three strands, respectively. Besides the strand parts in contact with the shell (outer areas and bottom) and contact points between the network layers (Figure 7d), the inlays were fully surrounded by air.

The drug-eluting surface area was calculated assuming an ideal square shape of the strands and was experimentally determined from the captured data (Table 2). The calculated surface area was lower compared to the true surface area determined via X-ray computed tomography with the exception of implants with a pore size of 0.4 mm. This is mainly because of deviations from the ideal square shape of the printed networks. In addition, the implants had a rough surface structure, which resulted in larger surfaces (Figure 7). We assume that the reason for the lower true surface area of implants with a pore size of 0.4 mm is that the weight of the upper layers led to a flattening of the layers below. This was the case especially at the contact points, further minimizing the pore size. This effect has a higher impact on small pore sizes compared to large pore sizes. Small pores also result in a higher density of the implant grid, likely reducing the cooling rate due to reduced air convection between the material and prolonging the time in which the material can deform plastically.

Theoretically, the surface area should decrease with a decreasing strand width or increasing pore size. The implant 0.4 × 0.4 should have the highest and the implant 1.2 × 1.2 the lowest surface area. However, the measurements showed a different order. For a strand width of 0.8 mm, the lowest surface area was determined for the 0.8 × 0.4 inlay, likely for the reasons mentioned above. Interestingly, the same value was determined for the implants 0.4 × 1.2 and 0.8 × 1.2 mm.

To print a representative drug module, approximately 7.5 cm of filament is required. Three sections of filaments (~2.5 cm, approximately ~20 mg TA) with a diameter of 1.719 ± 0.019 mm have a combined theoretical drug-eluting surface area of 4.12 cm^2^. Thus, implants have a 2- to 5-fold higher surface area compared to filaments, which should lead to an acceleration of TA release from the EC-based matrix.

### 3.2. Long-Term Dissolution Studies of Implants

**Figure 8:**
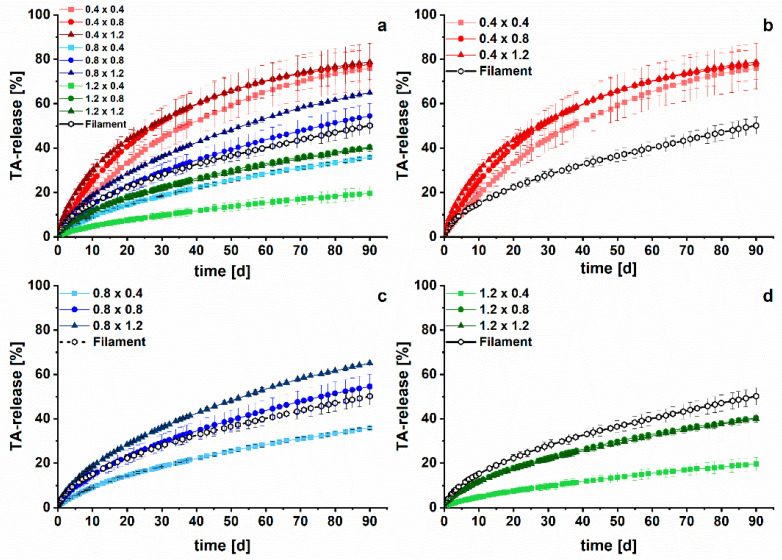
In vitro drug release of TA-loaded implants with different strand widths (first number) and varying pore sizes (second number) over three months (n = 3, mean ± s)*. (***a**) Overview of the nine different implants; (**b**) implants with a strand width of 0.4 mm, (**c**) 0.8 mm, and (**d**) 1.2 mm in comparison to the filament.

The produced implants were tested in vitro in long-term dissolution studies to examine the TA release performance of the different networks with varying strand widths and pore sizes. The dissolution data of an investigation period of three months are depicted in Figure 8. For the implants, the absolute drug content was calculated based on the mass of the drug-containing modules and the recovered content of filament F1 (Table 2). For the investigated filaments, the absolute drug content was experimentally determined by dissolving the filaments after terminating the dissolution in ethanol and measuring the drug content via HPLC. It has to be assumed that the calculated content of the implants is less precise than the content of the filaments.

In no case was 100% of the drug released. A range from 20 to 80% of relative release was realized, depending on the strand width/pore size combination, showing a high individualization degree via 3D printing from the same feedstock material. Over the first five days, a prolonged burst effect for all implants was observed, which might be beneficial to quickly reduce inflammatory-associated pain. This quick release was most likely caused by the TA located at the surface of the implants. After the initial burst, the release rate decreased. Implants with larger strand widths and smaller pore sizes tended to release TA slower from the non-erodible, pore-forming EC-HPMC matrix. This can be explained by the prolonged diffusion path lengths of the drug molecules, which consequently reduced drug diffusion and dissolution into the medium. The relative TA release took place in the order of the specific surface area (Figure 8) as expected.

Surprisingly, the filaments showed a dissolution behavior compared to the 0.8 × 0.8 implants, although the specific surface area was considerably lower with ~20 cm^2^/g. This shows the high impact of the unidirectional drug release from the implant system, limiting medium perfusion. Especially at the lowest pore size, a local saturation within pores might reduce TA dissolution.

Clinically relevant doses between 8.58 ± 1.38 mg (1.2 × 0.4) and 21.93 mg ± 1.31 mg (0.4 × 0.4) were released from the implants within three months of the test. In addition to the implant 1.2 × 0.4, the released absolute drug amount increased compared to the filaments after 90 days (8.97 ± 0.47 mg). The implant 1.2 × 0.4 combines both a small surface area and the narrowest pores, resulting in the slowest absolute drug release. With the developed approach, a successful modification and acceleration of TA release compared to filaments was facilitated without changing the formulation.

The drug release from the commercially available Volon^®^ A crystalline suspension was also determined to compare the drug release of the implants with the currently available formulation. Unfortunately, Volon^®^ A could not be tested in the same dissolution setup because of its fast dissolution and low solubility, which made it impossible to maintain sink conditions in the available setups. The low volume of the novel dissolution setup would have required a medium change within seconds, followed by additional medium changes over the course of dissolution. Therefore, dissolution was performed using a conventional dissolution setup in 1000 mL HEPES buffer. This renders a direct comparison of dissolution data difficult but allows us to draw some general conclusions.

**Figure 9:**
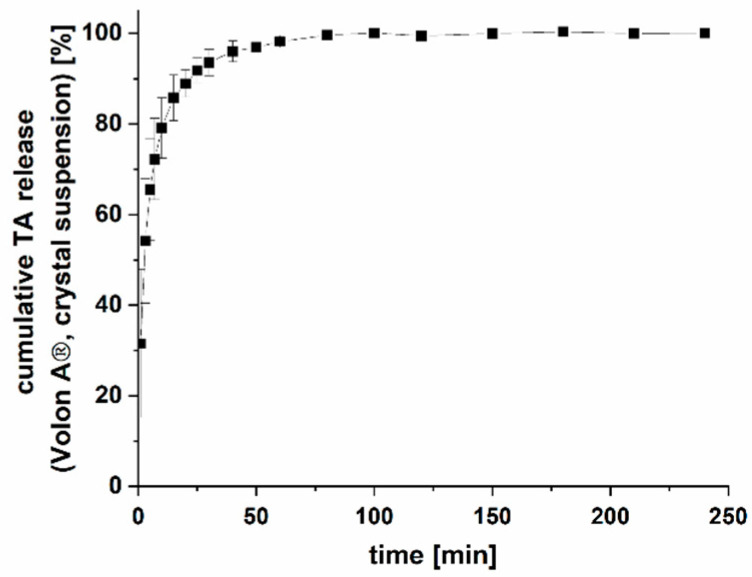
Drug release of a marketed TA crystal suspension Volon^®^ A (n = 3, mean ± s).

Overall, a strong retardation of the TA release was achieved with the implants compared to the marketed Volon^®^ A 10-5 mL for i.art. application. As seen in Figure 9, TA as a crystal suspension (6 mg, n = 3) was completely dissolved within 100 min, which is in line with the results from the literature [28,62]. The same amount was released within 13 to 40 days from implants, depending on the strand width/pore size combination of the network inlay. However, these results cannot be correlated to in vivo performance, as the crystal suspension has a residence time of approximately three days within the articular cavity [63].

**Figure 10:**
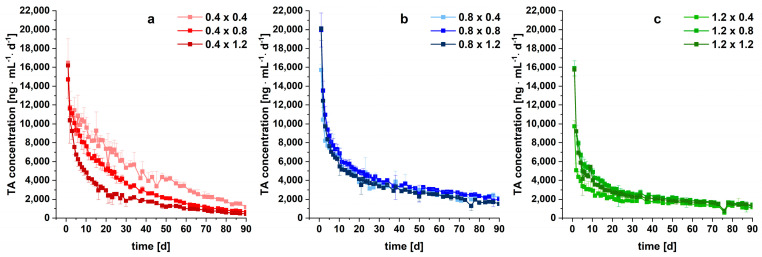
Daily TA release of 3D printed implants with (**a**) 0.4 mm, (**b**) 0.8 mm, (**c**) 1.2 mm strand widths and varying pore sizes (n = 3, mean ± s). Values are normalized to a volume of 50 mL.

To evaluate the daily TA release, a synovial volume of 50 mL was assumed. In healthy adults, the fluid amount is typically below 10 mL, but in inflammatory states, 25–75 mL are measured [64]. Figure 10 shows that the initial daily TA concentration was higher for implants with 0.8 mm strand width and a pore size of ≥0.8 mm. Consequently, the TA concentration within the articular cavity might be individualized.

Within the first month, a distinct decrease in the daily TA concentration for all implants was observed. Afterwards, almost constant TA levels between 444 and 2121 ng mL^−1^ d^−1^ after 90 days were observed, which is beneficial for maintenance treatment. As mentioned in the introduction, the PLGA-based microsphere extended-release formulation Zilretta* (FX600) was recently approved and showed improved therapy efficiency compared to the TA-loaded crystal suspension. Comparing the data with the results of in vivo study by Kraus et al. [28], considerably increased daily TA concentrations were achieved after three months with the implant 0.8 × 0.8 (implant 0.8 × 0.8 = 2030 ng/mL vs. FX 600 = 0.3 ng/mL). Hence, next to the benefits of the customization of the newly developed implant approach using 3D printing, further prolonged residence times and higher local concentrations might be realized. However, as the synovial clearance was not considered during in vitro long-term dissolution studies, a link to the in vivo performance is hardly possible. Amongst other pharmacokinetic conditions, systemic absorption, synovial fluid volume, as well as composition have a high impact on synovial concentrations [65]. In the future, in vivo-in vitro studies in combination with clinical endpoints are necessary for full-scope evaluation in humans.

The presented results demonstrate the capabilities of 3D printing to produce sophisticated customizable implants with a high degree of modification. A strong sustained release of TA was enabled to potentially prolong the residence time and likely increase the therapy efficiency of TA within the articular cavity compared to a marketed crystal suspension. Simultaneously, an individual adaption to anatomical structures is enabled by the presented modular implant concept.

### 3.3. Kinetic Analysis

Dissolution curves were subjected to a kinetic analysis according to Korsmeyer’s and Peppas’ approach to determine the underlying release mechanism via the diffusional release exponent n (Equation (1)) [49]. A release exponent of n = 0.45 represents a $\sqrt{t}$-kinetic, and n = 1 represents a zero-order-kinetic for cylindrical samples [49,66]. The drug-containing matrix consisted primarily of a non-erodible polymer (ethyl cellulose) and 25 wt% of a soluble polymer (HPMC). The soluble polymer acts as a pore former, which can influence the diffusional release exponent [67], and we expected a mix-kinetic dominated by the $\sqrt{t}$ characteristics of the non-erodible matrix.

The data were fitted using Equation (2). Values >5% of drug release were considered to cover the linear range, as TA was liberated comparatively quickly within the first five days. The obtained diffusional exponents as the slope of the linear regression of the 3D printed implants are shown in Table 3. Values varied between 0.58 and 0.70, which indicates an anomalous (non-Fickian) transport (0.45 < n < 1.0) [66]. A problem with this evaluation is that the above-mentioned values of n denoting a $\sqrt{t}$- or zero-order-kinetic are only valid for cylindrical samples. Even though similar threshold values exist for other geometries, none of them resemble the network structures of the implants in this study, which are additionally confined to unidirectional release. It has also been reported that small pore sizes of 3D printed network structures can limit hydrodynamics, thereby modifying the drug release further [15].

### 3.4. Modelling and Prediction of Drug Release

To model and potentially predict the TA release from implants, next to the Higuchi equation for porous, planar matrices (Equation (3)), different mathematical models (Peppas–Sahlin and Weibull) were fitted according to the approach of Windolf et al. [50]. No clear relationships between the variables of the Peppas–Sahlin and Weibull equations and the surface area to volume ratios were found. Predictions of the drug release were not possible, as the aforementioned characteristics of the modular implant system deviated too far from the boundary conditions of the release models.

Although the kinetic analysis indicated some deviations from a $\sqrt{t}$-kinetic, the fitting of dissolution curves with the Higuchi model (Equation (3)) led to acceptable results for implants (R^2^ = 0.9355–0.9929, Table 4), indicating that the model may be used for analysis. This represents a simple approach to describe the release behavior of porous, non-erodible planar matrix systems with suspended API, as in the case of printed implants [51]. Hence, the simplified Higuchi Equation (4), already evaluated by Korte und Quodbach for printed dosage forms with varying infill density, was examined to predict the dissolution curves of newly TA-loaded parenteral dosage forms [15].

The D_k_ of the implants was determined as the slope of the Higuchi plot (Q vs. $\sqrt{t}$). As all DDS were printed from the same starting material, it was initially expected that all implants would have the same D_k_—if the incorporated HPMC does not swell considerably during the release studies. In the case of HPMC swelling, we expected that the D_k_ of implants with a pore size of 0.4 mm is smaller as a potential clogging or narrowing of pores should lead to a diffusion barrier and limit medium perfusion [15]. In Table 4, the calculated D_k_ and the respective coefficient of determination for the linear fits of the implants are shown. D_k_ was interestingly only comparable for implants with the same strand width and a pore size ≥0.8 mm. Hence, the impact of the swelling of HPMC on D_k_ seems to depend on the pore size and strand width. The diffusion coefficient (D) is, consequently, time-dependent and not constant [68]. Contrary to the expectations, the D_k_ values of the implants 0.4 × 0.4 and 0.8 × 0.4 were larger.

One reason for this might be the single determination of the drug-eluting surface area, which is potentially not representative. Additionally, the drug-free shell was subtracted manually during image processing to determine the surface area. Both most likely led to inaccuracies in the D_k_ determination, predominantly for implants with many distinct small surfaces. In addition, the linear fits were the worst for implants with pore sizes of 0.4 mm.

Although D_k_ differed for the different strand widths, it was evaluated whether a prediction was possible. Data from implants with a pore size of 0.4 mm was excluded. For each strand width, the drug release of the TA-loaded implants was predicted using the measured drug-eluting surface area and D_k_ of the other pore sizes (Table 5).

**Figure 11:**
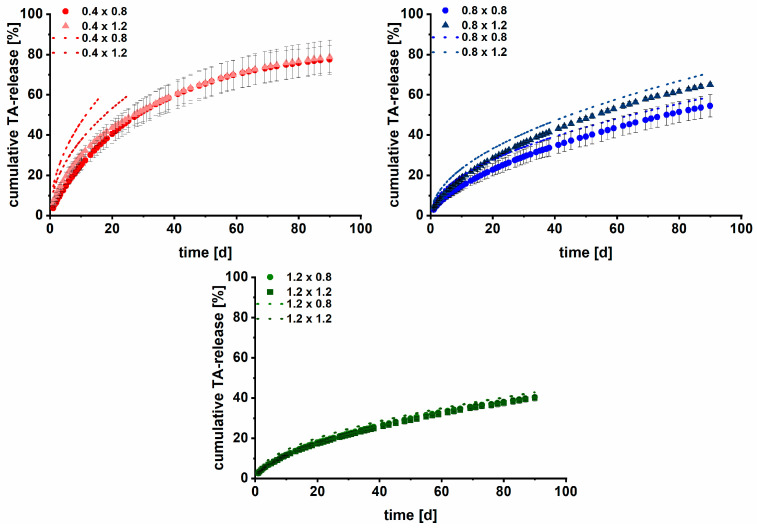
Observed (n = 3, mean ± s; 37 ± 0.5 °C, 200 rpm, 50–75 mL HEPES pH = 7.4) and predicted dissolution curves (dotted line) for printed implants with different strand widths (first number; 0.4 mm = red; 0.8 mm = blue; and 1.2 mm = green) and varying pore sizes (second number).

Figure 11 shows the observed versus predicted dissolution profiles of the different implants. In Table 5, the respective RMSEPs are listed to evaluate the goodness of the prediction. RMSEPs < 5% were considered acceptable. The predictive performance was not sufficient for implants with a strand width of 0.4 mm (RMSEP > 5%). The reasons are most likely the high impact of HPMC swelling at lower strand width and the surface area determination with only n = 1. Further, the pore size was always 0.4 mm in the z-direction (Figure 4, III), resulting in non-uniform medium perfusion and thus a deviating dissolution mechanism. It was not possible to image additional implants before the end of the study to increase the sample size for the surface area determination. This will lead invariably to inaccuracies in the data.

For implants with strand widths of 0.8 mm and 1.2 mm, the prediction was feasible (RMSEP ≤ 4.67%). The best results were obtained for implants with a strand width of 1.2 mm (2.32% and 2.65%). Surface changes due to swelling of the pore former HPMC were less pronounced relatively, leading to better results. Although the model is very sensitive towards small changes, a prediction based on the Higuchi equation is a promising and simple approach to predict the drug release of the 3D printed matrix implants. It is especially advantageous that only one dataset per strand width is required for prediction, resulting in less work during product development and quality control. A prediction with sufficient goodness is possible for one strand width and different pore sizes. The applicability, however, is limited to strand widths and pore sizes ≥ 0.8 mm. This might be extended by using non-swelling pore formers (e.g., polypropylene glycoles).

**Figure 12:**
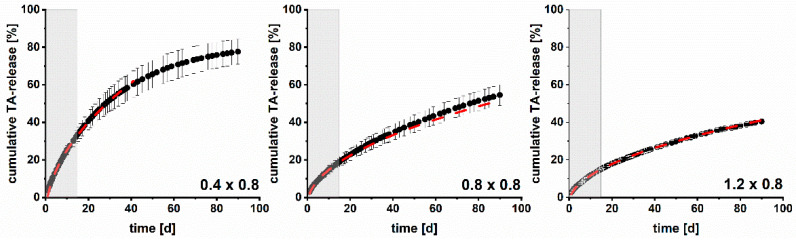
Exemplary observed (black dots, n = 3, mean ± s; 37 ± 0.5 °C, 200 rpm, 50–75 mL HEPES pH = 7.4) and predicted (red dotted line) dissolution curves for 3D printed implants with varying strand widths and a pore size of 0.8 mm based on drug release data of the initial 15 days (grey zone = used experimental data for prediction).

A downside of the above-mentioned method is that dissolution data over a long dissolution time are required to determine D_k_. In order to reduce the analytical time required for future studies of such implants, the Higuchi model was tested in a different way as well. Based on the release data obtained during the initial 15 days, it was examined if a prediction of the remaining 75 days was possible. Therefore, the relative drug release of the respective implant was plotted as a function of $\sqrt{t}$. The obtained linear regressions (R^2^ shown in Table 5) were used to determine the drug release up to the residual investigation period. Exemplary observed and predicted dissolution curves (maximum until 60% drug release) of implants with a pore size of 0.8 mm and varying strand widths are shown in Figure 12. The corresponding RMSEPs are listed in Table 5. The predictive performance up to 60% drug release was good for all implants (RMSEPs ≤ 3.15%). The results are promising to drastically reduce experimental work to two weeks for future studies during medicinal product development and quality control for customized parenteral DDS.

## 4. Conclusions

In this study, an innovative, modular, two-compartment implant design was presented and successfully realized using CAD and FDM printing. With this approach, it is possible to individualize shape, dosage, and drug release independently. Additionally, a simple approach to predict the drug release by changing the strand width/pore size combination was introduced. Both are essential to facilitate the on-demand production of customized drug-loaded implants for various diseases in hospitals with predictable dissolution behavior of the parenteral depot system. Therefore, the therapy efficiency of current parental therapy options may be enhanced and systemic side effects may be reduced. The limitations of conventional manufacturing methods of parenteral depot systems with respect to individualization can be overcome. Comparative in vivo studies with clinical endpoints are necessary to investigate the performance of such implants in animals and humans.

To enhance the mechanical resilience of the implant shell, as it may serve as a replacement for anatomical structures, other materials for the shell, e.g., polyether ether ketone, may be favored. It may also be possible to realize the implant in combination with titanium, a material commonly used for prostheses. Due to an observed fragility of the drug-containing inlay, a three-compartment design would have to be designed. The two compartments of the presented drug-free shell and embedded drug-loaded network can be printed and then combined with a titanium prosthesis with a prefabricated cavity. Further investigations of the overall implant stability under mechanical stress are necessary.

The prediction of drug release was partially feasible based on the Higuchi model using D_k_ and the determined drug-eluting surface area. Restrictions are caused by kinetic changes due to non-uniform medium perfusion at low pore sizes, the impact of the pore former HPMC, and the unidirectional drug release from the implants. Therefore, further studies should focus on different mathematical models in combination with different materials, e.g., non-swellable pore formers, to circumvent those limitations. However, a prediction of the long-term dissolution performance based on the data from the initial 15 days was shown. This simplifies the evaluation and quality control of individualized parenteral depot systems and reduces overall analytical effort.

## Figures and Tables

**Table 1:** Composition of filaments for the printing of two-compartment implants [% *w*/*w*].

Substance		TA	EC	TEC	HPMC	Fumed Silica
	Function	API	Thermoplastic Polymer	Plasticizer	Pore Former	Glidant
Formulation						
F1 (drug-loaded implant inlay)		10	54.64	10	25	0.36
F2 (drug-free shell)		-	89.64	10	-	0.36

**Table 2:** Physical properties of module II (TA-loaded implant inlay): dimensions and mass, TA dose (n = 3), the calculated and true drug-eluting surface area (A, n = 1), their ratio, and the specific surface area (SSA).

	Implant								
Strand width [mm]	0.4	0.4	0.4	0.8	0.8	0.8	1.2	1.2	1.2
Pore size [mm]	0.4	0.8	1.2	0.4	0.8	1.2	0.4	0.8	1.2
Dimensions (x, y) [mm]	14	14	14	14.8	15.2	15.2	13.2	12.8	13.2
Height [mm]	3.2	3.2	3.2	3.2	3.2	3.2	3.6	3.6	3.6
Mass [mg] (mean ± s)	327.0 ± 21.5	222.1 ± 16.0	146.4 ± 19.9	517.3 ± 18.3	385.12 ± 28.8	272.1 ± 10.3	491.4 ± 7.7	241.7 ± 4.7	316.0 ± 9.7
TA [mg] (mean ± s)	29.1 ± 1.9	19.7 ± 1.4	13.0 ± 1.8	45.8 ± 1.6	34.1 ± 2.6	24.1 ± 0.9	43.5 ± 0.7	30.5 ± 0.4	28.0 ± 0.9
A_cal_ [cm^2^]	23.02	16.93	12.45	15.78	13.31	10.89	10.25	8.06	7.27
A_true_ [cm^2^]	20.54	18.54	13.08	12.95	15.30	13.08	9.80	8.69	7.91
A_true_/A_cal_ [-]	0.89	1.10	1.05	0.82	1.15	1.20	0.96	1.08	1.09
SSA [cm^2^/g]	62.6	83.5	89.3	25.0	39.7	48.1	19.9	25.4	25.0

**Table 3:** Determined diffusional exponents n and coefficients of determination of implants according to Korsmeyer’s and Peppas’ approach.

	Implant								
Strand width [mm]	0.4	0.4	0.4	0.8	0.8	0.8	1.2	1.2	1.2
Pore size [mm]	0.4	0.8	1.2	0.4	0.8	1.2	0.4	0.8	1.2
n [mean ± s]	0.76 ± 0.02	0.69 ± 0.05	0.54 ± 0.01	0.66 ± 0.02	0.68 ± 0.03	0.60 ± 0.01	0.65 ± 0.01	0.59 ± 0.02	0.58 ± 0.00
R^2^	>0.9933	>0.9924	>0.9887	>0.9952	>0.9991	>0.9992	>0.9986	>0.9964	>0.9988

**Table 4:** D_k_ of 3D printed implants obtained from Higuchi plots and R^2^ of linear fits.

Implant (Strand Width × Pore Size)	$D_{k}=\frac{Q}{\sqrt{t}}$ [mg·cm^−2^·d^−0.5^] Based on Measured A	R^2^
0.4 × 0.4	0.1401	0.9935
0.4 × 0.8	0.1182	0.9977
0.4 × 1.2	0.1031	0.9981
0.8 × 0.4	0.1460	0.9988
0.8 × 0.8	0.1386	0.9993
0.8 × 1.2	0.1371	0.9994
1.2 × 0.4	0.1005	0.9952
1.2 × 0.8	0.1592	0.9997
1.2 × 1.2	0.1575	0.9994

**Table 5:** Data of the respective prediction of the drug release of different implant networks using the Higuchi model.

Implant (Strand Width × Pore Size)	Prediction Based on D_k_ and the Determined Surface Area According to [15]		Prediction Based on Initial Drug Release Data until Day 15	
	Data Set Used for Prediction	RMSEP [%]	R^2^	RMSEP [%]
0.4 × 0.4	-	-	0.9974	2.19
0.4 × 0.8	0.4 × 1.2	19.44	0.9989	0.62
0.4 × 1.2	0.4 × 0.8	8.24	0.9999	3.15
0.8 × 0.4	-	-	0.9982	0.86
0.8 × 0.8	0.8 × 1.2	4.29	0.9989	1.85
0.8 × 1.2	0.8 × 0.8	4.67	0.9998	1.21
1.2 × 0.4	-	-	0.9988	1.41
1.2 × 0.8	1.2 × 1.2	2.32	0.9979	0.21
1.2 × 1.2	1.2 × 0.8	2.65	0.9962	0.57

## Data Availability

Not applicable.

## Supplementary Materials

The following supporting information can be downloaded at: https://www.mdpi.com/article/10.3390/pharmaceutics15082097/s1, Figure S1: Technical drawing of the modular implant concept, consisting of the drug-free shell (grey) and the drug-loaded network (red); Table S1: HME barrel temperatures and haul-off speed applied during filament production; Table S2: Detailed 3D printing settings for two-component implants; Table S3: Exemplary true strand widths [µm] of implants networks (determined via X-ray computed tomography (n = 3).
