# Supplementary material for: Customizable 3D Printed Implants Containing Triamcinolone Acetonide: Development, Analysis, Modification, and Modeling of Drug Release
Source: Pharmaceutics. 2023 Aug 8;15(8):2097. doi: 10.3390/pharmaceutics15082097 (PMC10459585; doi:10.3390/pharmaceutics15082097)
Supplement: Supplementary file 1 [file pharmaceutics-15-02097-s001.zip › pharmaceutics-2317108-supplementary.pdf]

pharmaceutics

Supplementary Materials: Customizable 3D-printed Implants Containing Triamcinolone Acetonide: Development, Analysis, Modification, and Modeling of Drug Release

Hanna Ponsar, Julian Quodbach

Supplementary information:

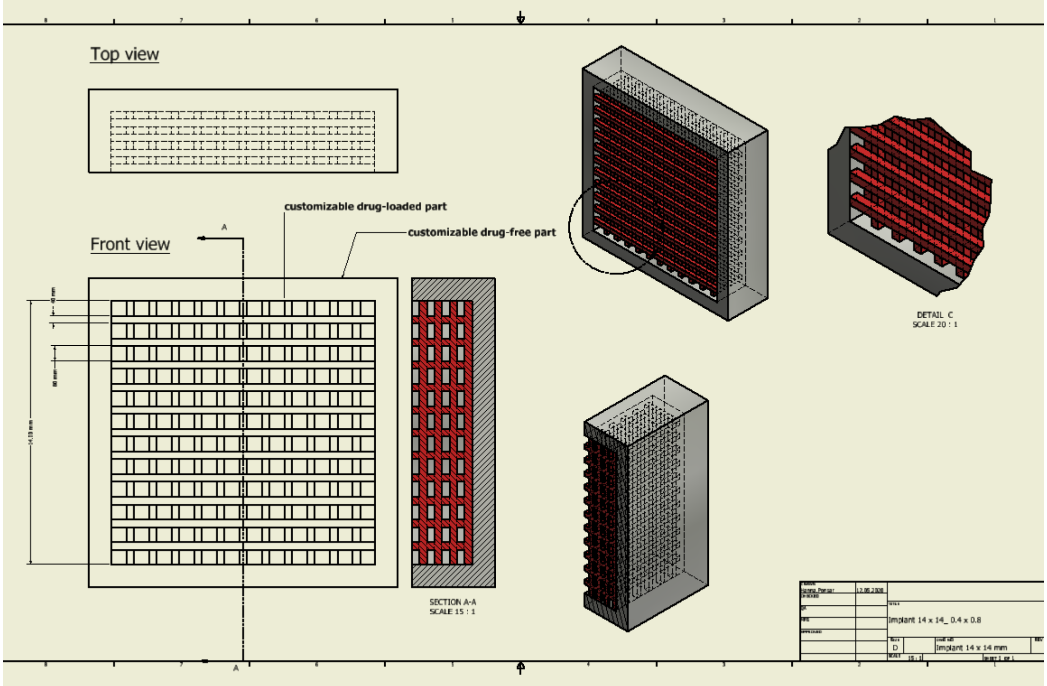

Figure S1. Technical drawing of the modular implant concept, consisting of the drug-free shell (grey) and the drug-loaded network (red).

Table S1. HME barrel temperatures and haul-off speed applied during filament production.

Formulation	Barrel temperature from zone T1 (gear) to T10 (die) [°C]										Haul-off speed [m/min]
	T1	T2	T3	T4	T5	T6	T7	T8	T9	T10	
**F1, F2**	-	30	80	120	170	180	180	190	190	190	2.1

Table S2. Detailed 3D-printing settings for two component implants.

Settings	Two-component implants			
	Drug-free shell	Drug-loaded network inlays		
		0.4 mm	0.8 mm	1.2 mm
**Filament**	F6	F12	F12	F12
**Print temperature [°C]**	185	185	185	185
**Build plate temp. [°C]**	90	90	90	90
**Nozzle diameter [mm]**	0.4	0.4	0.4	0.4
Layers and perimeters				
**Layer height (first layer height) [mm]**	0.2 (0.2)	0.2 (0.2)	0.2 (0.2)	0.2 (0.2)

Settings	Two-component implants			
	Drug-free shell	Drug-loaded network inlays		
		0.4 mm	0.8 mm	1.2 mm
**Perimeters**	1	0	1	1
**Solid layers (top and bottom)**	0	0	0	0
**Seam position**	nearest	nearest	nearest	nearest
Infill				
**Infill density [%]**	100	100	100	100
**Fill pattern**	rectilinear			
**Fill angle [°]**	45	90	90	90
Speed [mm/s]				
**Perimeters**	25	10	20	15
**Infill**	20	8	16	12
**Travel**	80	120	80	120
**First layer**	12.5	10	10	15
Others				
**Extrusion width [mm]**	0.39	0.39	0.4	0.4
**Infill/perimeter overlap [%]**	5	N/A	5	5
**Ooze shield**	yes	yes	yes	yes

Table S3. Exemplary true strand widths [µm] of implants networks (determined via X-ray computed tomography (n = 3)).

Implant (strand width x pore size)	Strand width (mean ± s) [µm]	Deviation from set point
0.4 x 0.4	429 ± 21	0.07
0.8 x 0.8	914 ± 37	0.13
1.2 x 1.2	1241 ± 61	0.03
